# Carotid Floating Thrombus With Patent Foramen Ovale: An Unusual Cause of Stroke in Young

**DOI:** 10.7759/cureus.75034

**Published:** 2024-12-03

**Authors:** Aniruddha S Jog, Shashank Nagendra, Pranay Bandgar, Karthik S Goud

**Affiliations:** 1 General Internal Medicine, Grant Government Medical College, Mumbai, IND; 2 Neurology, Grant Government Medical College, Mumbai, IND

**Keywords:** carotid free-floating thrombus, digital subtraction angiography(dsa), patent foramen ovale (pfo), stroke, young adult male

## Abstract

Embolic strokes of undetermined source (ESUS) form a quarter of all ischemic strokes, with the majority of them being thromboembolic. Carotid free-floating thrombus (CFFT) is a rare cause of ischemic stroke. There are no current guidelines outlining the management of CFFT. Patent foramen ovale (PFO) is a common cardiac abnormality that is generally not considered to be an independent risk factor for stroke. But, in certain stroke cases involving younger patients (<55 years old), PFO may be a source of paradoxical embolism. However, ipsilateral atherosclerosis in carotids has a low prevalence in patients with clinically significant PFO. We present an unusual case of stroke with two possible etiologies, CFFT, and a large PFO, begging the question, which lesion was the culprit?

## Introduction

Ischemic strokes in almost a quarter of cases are cryptogenic (unknown cause) [1]. Embolic stroke of undetermined source (ESUS) constitutes approximately 25% of all strokes of ischemic etiology. Evidence suggests that most of these strokes are thromboembolic [1]. Emboli originate from various potential sources, like cardiac or arterial. Patent foramen ovale (PFO) is a common cardiac abnormality found in about 25% of subjects in the general population and does not seem to be an independent risk factor for stroke [2]. However, some studies and case reports suggest that PFO associated with ischemic stroke in young patients (<55 years) should not be considered incidental [3,4]. We present an unusual case of stroke in a young man with an intraluminal floating carotid thrombus with an incidentally detected PFO. 

## Case presentation

**Figure 1:**
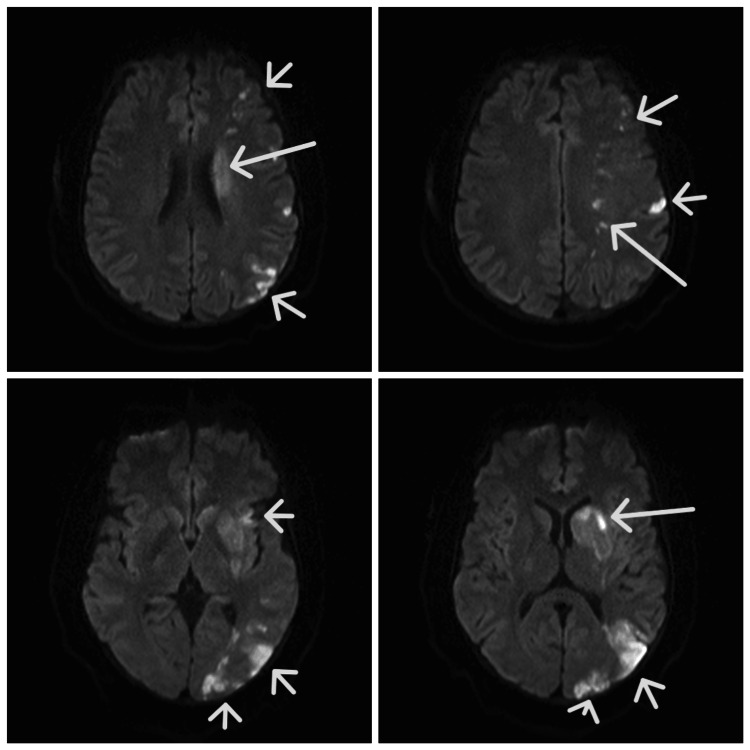
MRI brain DWI showing left MCA territory infarct (see arrows) DWI: Diffusion-weighted Image, MCA: Middle cerebral artery

**Figure 2:**
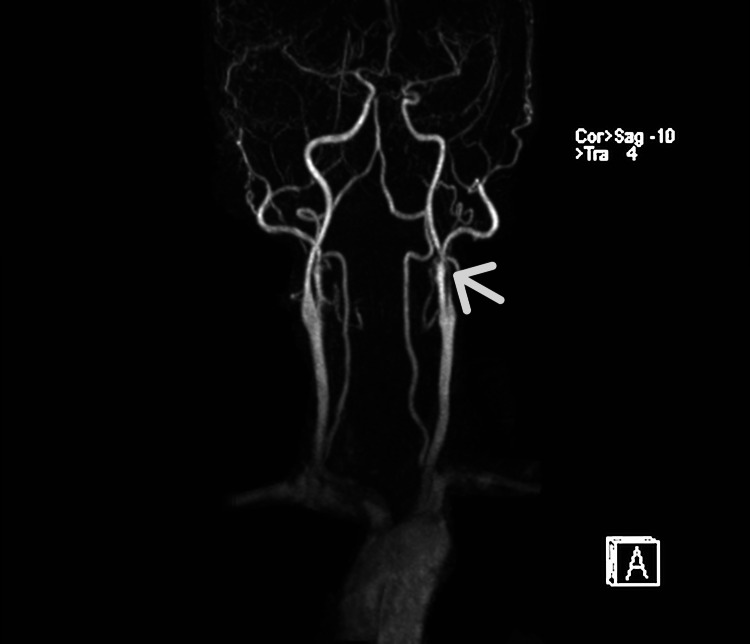
MR angiogram showing left ICA narrowing (arrow) ICA: Internal carotid artery

A 36-year-old man presented with sudden onset right-sided weakness and aphasia of 12 hours. He had a similar episode two days before that lasted for 10 minutes and resolved without any medical treatment. His past medical history was unremarkable, with no diabetes, hypertension, or heart disease. There was no significant history of stroke in the family. He was an active smoker, smoking 1-2 cigarettes per day for around 10 years. On examination, the patient was conscious and oriented to time, place, and person. He had upper motor neuron facial weakness on the right side. Power in the right upper and lower limbs was 3/5 (MMRC) proximally and distally. The patient had motor aphasia. NIHSS score was 12. In this regard, the patient was advised to have an MRI of the brain with an angiogram urgently. This showed left middle cerebral artery (MCA) territory scattered embolic non-hemorrhagic infarcts (Figure 1). The left internal carotid artery (ICA) showed severe narrowing (80-90%) distal to its origin (Figure 2).

**Figure 3:**
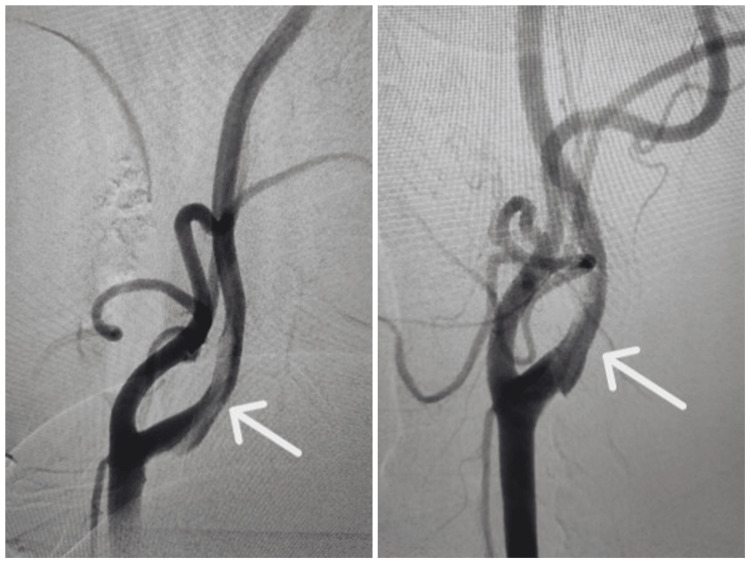
4 Vessel DSA showing a floating thrombus in the left ICA (arrows) ICA: Internal carotid artery

The remaining investigations, including full blood count, renal profile, blood sugar levels, and electrocardiogram, were normal. LDL cholesterol level was 121 mg/dl. Trans-thoracic echocardiography was normal; however, trans-esophageal echocardiography was advised nonetheless. A 24-hour Holter was done and unremarkable. Given clinical and MRI findings, the patient was started on dual antiplatelets (aspirin and clopidogrel) and atorvastatin. A 4-vessel Digital Subtraction Angiography (DSA) was done to examine the irregularity in the left carotid. This showed a floating thrombus in the left ICA (Figure 3).

**Figure 4:**
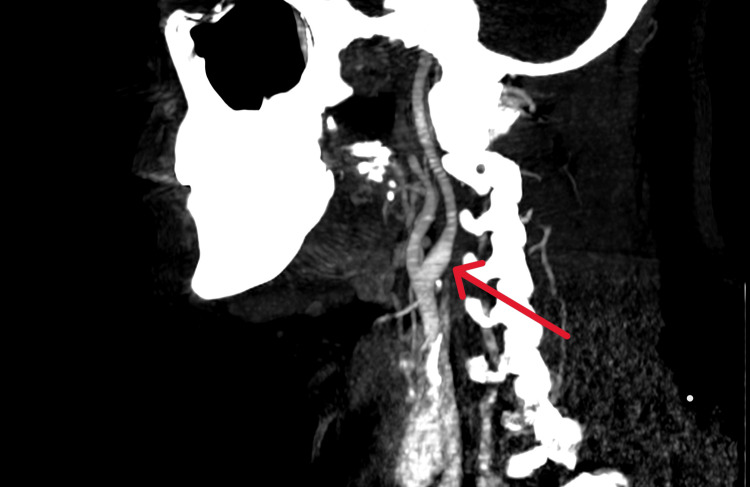
CT angiogram after one week of therapy showing subtle wall thickening in the left ICA without any visible filling defect (red arrow) ICA: Internal carotid artery

In view of this, the patient was started on therapeutic anticoagulation and was treated with heparin and aspirin. Repeat imaging at seven days was planned. Other investigations that included serum homocysteine levels were raised at 45 mmol/L. However, MTHFR mutation was not detected. Vasculitis screen, including anti-nuclear antibody, anti-phospholipid antibody screen, and ANCA screen, were negative. The patient was planned for a thrombophilia workup at a later stage after a discussion with hematology. Repeat CT brain with angiogram done after 1 week showed subtle wall thickening without any web or dissection. However, no thrombus or filling defect was visible (Figure 4).

The patient was thus discharged on apixaban, aspirin, and statin. At the follow-up appointment, TOE with air bubble contrast showed a large right-to-left shunt across PFO. The Risk of Paradoxical Embolism (RoPE) score in this case was 8. However, given the obvious finding of free-floating thrombus in the carotid, PFO closure was not recommended. The patient has been followed up in the neurology clinic and has no recurrent complaints. We have recommended continuing apixaban for one year. We aim to continue a single antiplatelet agent after this and discontinue apixaban if the thrombophilia screen is negative.

## Discussion

Evaluation of stroke requires a combination of thorough clinical examination and imaging. The purpose of the evaluation is to find a cause, as treatment can be tailored to the etiology, which may include different modalities, especially if there is a coexistence of two pathologies. This case was interesting as there were two possible causes of stroke, the carotid intraluminal floating thrombus and an incidentally detected large patent foramen ovale (PFO).

In most patients, the etiology of an ischemic stroke is embolic. Most of these emboli are thrombotic, with the source being arterial, venous, or from heart chambers and valves [5]. Carotid free-floating thrombus (CFFT) is a rare cause of embolic stroke, contributing to around 1.6% of cases of stroke [6]. In spite of being relatively uncommon, CFFTs are high-risk lesions with an 11% stroke risk or death within 30 days of detection [6]. Etiology in nearly 80% of cases of CFFT with TIA/stroke is atherosclerotic plaque rupture [6,7]. Moreover, around 50% of cases have significant carotid stenosis, which makes them candidates for urgent revascularization [7]. The remaining 20% of cases had non-atherosclerotic etiologies such as cardioembolism, carotid dissection, arrhythmias, malignancy, and hypercoagulability [6]. Of the non-atherosclerotic etiologies, around 4% of the patients are embolic strokes of undetermined source (ESUS) [7]. The fate of a CFFT, like any thrombus, potentially includes propagation, embolization, or dissolution [8].

The diagnostic modality of choice in CFFT is DSA, however, due to ease during follow-up and the non-invasive nature of the investigation, CT angiogram and Carotid Doppler have become preferred modalities [6]. In our case, the patient had an MRI angiography as the first non-invasive investigation; however, we proceeded to DSA to further investigate the abnormality noted in the internal carotid artery. This showed a floating thrombus in the left CCA.

Cardiac abnormalities are prevalent in many patients who present with an embolic stroke. PFO is prevalent in about a quarter of the general population [2]. Although there are case reports of PFO being a causative factor for stroke, a prospective population-based study showed that PFO is not a risk factor for future stroke [2]. However, a few others say that PFO has been an associated finding in young strokes and hence should be actively sought. Moreover, if found, PFO in these cases should not be regarded as incidental [3]. The cause of stroke in PFO is presumed to be paradoxical embolism. RoPE score identifies stroke-related PFO in patients with cryptogenic stroke [9]. RoPE of >7 had a PFO-attributable fraction of over 71% while RoPE <=7 had a PFO-attributable fraction of 0% [10]. In our case, the patient had a RoPE of 8, suggesting that the chance of stroke being due to PFO was 84%. However, unlike our case, a study in patients with ESUS showed that ipsilateral carotid atherosclerosis is less prevalent in patients with a possible pathogenic PFO (RoPE >=7) [11]. This makes our case relatively rare.

The lack of consensus in the management of patients with CFFT makes the decision to treat such patients challenging. However, the risk of recurrent stroke in one prospective study was low when patients were treated with a combination antithrombotic regimen (heparin with one antiplatelet agent) [12]. In our case, we managed our patient on similar lines with seven days of heparin and low-dose aspirin. Repeat imaging after seven days did not show any intra-luminal thrombus. PFO closure was not recommended as we felt that clinically this stroke was related to the CFFT. The patient was discharged on a DOAC (apixaban), aspirin, and a statin. The patient has been advised to continue DOAC for one year. The patient has been followed up in the neurology clinic and has no fresh complaints.

## Conclusions

This case is unique in that it presents us with two possible etiologies of stroke at the same time. However, this begs the question: Can PFO cause a clot that will migrate to the carotid to cause a floating thrombus? Or is it a red herring, an innocent bystander? We believe that it is most likely the latter. Hence, we managed the patient with an anticoagulant and a single antiplatelet agent. Also, PFO closure was not recommended.
